# Dynamin inhibitor dynasore modulates longitudinal bone growth in a hormetic manner

**DOI:** 10.1186/s12915-026-02687-4

**Published:** 2026-07-16

**Authors:** Jose G. Marchan-Alvarez, Sanya Koikkara, Ruihan Zhou, Amal Nazaraliyev, Oscar P. B. Wiklander, Phillip T. Newton

**Affiliations:** 1 Department of Women’s and Children’s Health, Karolinska Institutet, Solna, Sweden; 2 Astrid Lindgren Children’s Hospital, Stockholm, Sweden; 3 Division of Biomolecular and Cellular Medicine, Clinical Research Center, Department of Laboratory Medicine, Karolinska Institutet, Stockholm, Sweden; 4 Breast Center, Karolinska Comprehensive Cancer Center, Karolinska University Hospital, Stockholm, Sweden

**Keywords:** Endochondral ossification, Dynasore, Autophagy, MTORC1, Bone elongation, Ex vivo metatarsal culture

## Abstract

**Background:**

Longitudinal bone growth occurs via endochondral ossification, involving a complex interplay of chondrocyte proliferation, differentiation, and matrix remodeling. As with all mammalian cells, chondrocytes require dynamin for mitochondrial fission, to shuttle vesicles from the Golgi apparatus, and for both clathrin- and caveolin-mediated endocytosis. Here, we aimed to test the functions of dynamin on bone growth. To do so, we applied dynasore—a small molecule that is a reversible dynamin inhibitor—to mouse metatarsal bones cultured ex vivo. We assessed gross changes using bone length measurements combined with EdU detection, immunostaining, super-resolution microscopy and transmission electron microscopy.

**Results:**

Dynasore induced a dose-dependent hormetic effect on bone elongation: while high concentrations (220 µM) impaired growth and abolished chondrocyte proliferation, low-dose treatment (40 µM) significantly increased longitudinal bone growth. Histological analysis demonstrated that low dose dynasore augmented epiphyseal cartilage expansion and matrix accumulation, while reducing chondrocyte proliferation. Immunostaining indicated that 40 µM dynasore preserved collagen type X synthesis, activated mTORC1 signaling, and blocked autophagy, based on SQSTM1 accumulation. Low dose dynasore treatment expanded the thickness of the filamentous actin layer at the plasma membrane and deepened endocytic pits containing collagen fibril-like electron-dense extracellular structures, indicating that impaired cartilage remodeling was associated with growth-associated matrix accumulation. Finally, the use of a structurally unrelated dynamin inhibitor, dynole, indicated that the effects of dynasore were partially mediated by its actions on dynamin.

**Conclusions:**

Dynasore exerts hormetic effects on epiphyseal chondrocytes, wherein low doses stimulate bone elongation, and high doses impair chondrocyte function.

**Supplementary Information:**

The online version contains supplementary material available at 10.1186/s12915-026-02687-4.

## Background

During childhood and adolescence, humans grow taller due to the elongation of bones such as those in our legs and spine. These bones grow through a process called endochondral ossification within the growth plate, a transient cartilaginous structure composed of chondrocytes arranged in distinct histological zones. The resting zone (RZ) contains progenitor cells that replenish the proliferative zone (PZ), where chondrocytes undergo rapid clonal expansion and form columnar stacks. These cells then progress into the pre-hypertrophic stage, where chondrocytes cease division, increase in size, and subsequently enter the hypertrophic zone (HZ), where they further enlarge, remodel and mineralize their hyaline cartilage extracellular matrix (ECM). Mineralized cartilage is then used as a scaffold on which new bone tissue is deposited by osteoblasts, ultimately contributing to bone elongation and height gain in children and adolescents [1].

The transition of chondrocytes through different morphological and functionals stages in the epiphyseal cartilage is regulated by several molecular players. These molecules participate in key regulatory pathways such as the fibroblast growth factor (FGF) and Notch signaling pathways, growth hormone (GH)-insulin-like growth factor 1 (IGF-1) axis, and the Indian hedgehog (IHH)-parathyroid hormone-related peptide (PTHrP) feedback loop [2, 3]. For such extracellular signaling pathways to act in chondrocytes, intracellular trafficking machinery is required to, for example, fine-tune the abundance, localization, and signaling activity of critical receptors. Thus, endosomal trafficking regulates receptor recycling versus degradation, thereby modulating the intensity and duration of pathway activity. Disruption of trafficking can potentially shift the balance between proliferative and differentiative cues. Therefore, the interplay of tissue architecture with key signaling pathways and receptor trafficking creates a highly coordinated system governing skeletal elongation.

The protein dynamin is crucial for certain forms of membrane scission that are conserved among higher eukaryotes [4]. Dynamin is a guanosine triphosphatase (GTPase) that is recruited into oligomeric helices that form around a lipid bilayer membrane tube. The dynamin oligomer then undergoes conformational changes that constricts the membrane tube, narrowing it to a point where the inner luminal radius falls below 2 nm, prior to fission of the membrane in a GTP-dependent manner [5]. Thus, dynamin is required for membrane trafficking processes, such as clathrin- and caveolin-mediated endocytosis [6], shuttling of vesicles from the Golgi apparatus as well as mitochondrial fission [7]. While its functions mean that dynamin is involved in a large number of cellular processes, our understanding of its roles in chondrocytes during bone growth is incomplete.

**Fig. 1:**
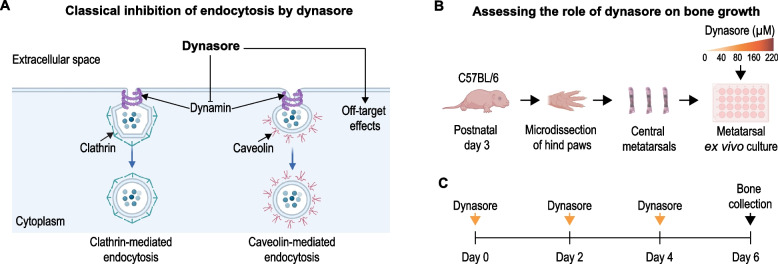
Experimental workflow. **A** Schematic overview of dynasore functions; whilst dynasore has been widely used as a dynamin inhibitor to block various endocytic pathways, including clathrin-mediated and caveolin-mediated endocytosis, less well-described dynamin-independent effects are also reported. **B**, **C** Central metatarsals were isolated from the hind paws of C57BL/6 mice and cultured ex vivo. Dynasore was then applied in a dose–response manner at days 0, 2 and 4

To pharmacologically inhibit dynamin function, dynasore is commonly used. This cell-permeable small molecule acts as a non-competitive, reversible inhibitor of dynamin. By binding to the GTP-binding domain, dynasore targets the GTPase activity of dynamin, thereby preventing the hydrolysis of GTP required for membrane fission [6]. The actions of dynasore in disrupting the scission of vesicles at the inner leaflet of the plasma membrane [8] (Fig. 1A), has been observed in different cell types such as immune cells [9], muscle cells [10], osteosarcoma cells [10], and articular chondrocytes [11]. Yet, beyond its classical role as a dynamin inhibitor, dynasore may alter other processes in a dynamin-independent manner, including cholesterol homeostasis, lipid raft integrity, lysosomal inhibition [12] and actin remodeling [7]. Whereas the properties of dynasore have been explored in the articular cartilage [11], the effects on endochondral ossification remain to be determined.

Hence, we aimed to test the effects of dynamin inhibition mediated by dynasore on longitudinal bone growth.

## Results

### Dynasore has hormetic effects on bone elongation ex vivo

**Fig. 2:**
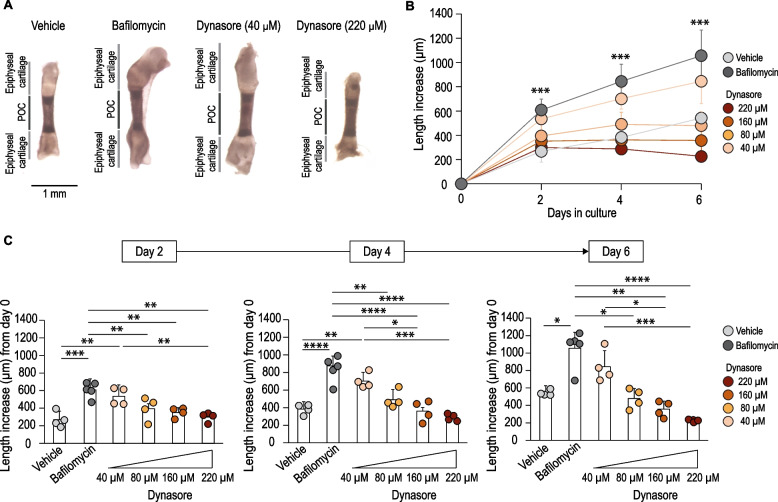
Dynasore leads to differential regulation of longitudinal bone growth. **A** Ex vivo cultures of metatarsals isolated from three-day old mice were maintained in the presence of vehicle, bafilomycin (positive control) and dynasore (treatment) for 6 days, as per Fig. 1C. Representative images at day 6. **B**, **C** Longitudinal bone growth was assessed every two days. POC: primary ossification center. *N* = 4 animals (12 bones) for vehicle, bafilomycin and dynasore. Data is presented as mean ± SD. Asterisks in panel B and C indicate statistically significant differences, *p < 0.05. **p < 0.01. ***p < 0.001. ****p < 0.0001

We used a well-established model to culture murine metatarsals ex vivo [13] and treated the bones with increasing concentrations of the inhibitor: 40 µM, 80 µM, 160 µM, and 220 µM (Fig. 1B-C). This dosage was based on previous studies reporting the use of dynasore, with concentrations of ≤ 80 µM typically achieving a strong inhibition of endocytosis [6, 9, 14]. Here, whilst the highest dose impaired normal growth, we were surprised to find that a dose of 40 µM stimulated longitudinal growth (Fig. 2A-C). Morphological changes caused by dynasore were evident from day 2 of culture, resulting in longer bones compared to both vehicle and higher concentrations (220 µM) by day 4 and 6 (Additional file 1: Fig. S1A; Fig. 2A-C). Of note, the lowest dose of dynasore (40 µM) supported robust bone growth to a similar extent as bafilomycin (Fig. 2A-C), a well-established promoter of metatarsal elongation [15]. On the contrary, 220 µM dynasore caused a marked reduction in bone length by the end of the culture period (Fig. 2A-C). These changes were also evident in the assessment of bone growth rate, which demonstrated that all tested dynasore concentrations initially promoted bone growth until day 2. However, in the subsequent days, the growth rate declined sharply across all conditions, except in bones treated with 40 µM dynasore, where longitudinal bone growth was sustained in a comparable manner to the bafilomycin treatment (Additional file 1: Fig. S1B).

To evaluate potential sex-dependent differences in response to dynasore, we carried out ex vivo cultures of male and female metatarsals. Of note, bones from male and female mice had a pronounced overgrowth in the presence of 40 µM dynasore compared to vehicle-treated controls (Additional file 1: Fig. S2A-C), thus indicating that the pharmacological effect of dynasore (40 µM) on longitudinal bone growth is robust and independent of sex.

### Dynasore alters bone growth through differential dose-dependent effects on chondrocytes

**Fig. 3:**
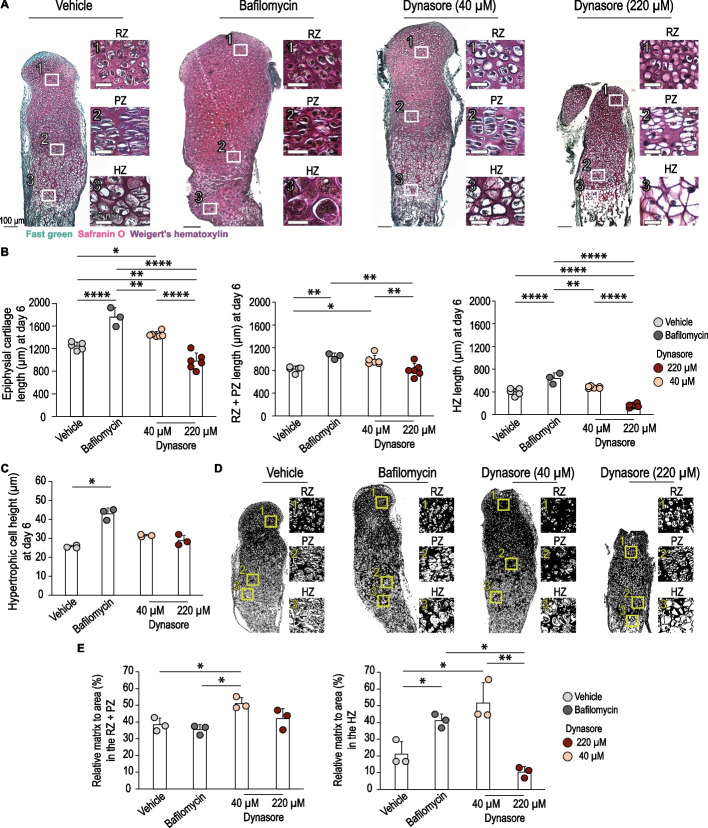
A low concentration of dynasore (40 µM) stimulates bone growth. **A** Histological characteristic of the epiphyseal cartilage: resting, proliferative, and hypertrophic zones stained with Safranin O/Fast Green. **B-C** Histomorphometric analysis of samples prepared as per panel A. **D** Masks displaying relative matrix present in samples were prepared using Image J. **E** Relative matrix to area assessment. RZ: resting zone; PZ: proliferative zone; HZ: hypertrophic zone. Data in B, C and E is presented as mean ± SD, *n* = 5 bones, except in bafilomycin, the hypertrophic cell height, and relative extracellular matrix/area, which represent 3 bones. *p < 0.05, **p < 0.01, and ***p < 0.001. ****p < 0.0001. Please note that metatarsals were culture for 6 days, as per Fig. 1C

To explore the changes induced by dynasore, we selected bones from the doses giving the strongest hormetic effects, 40 µM (which stimulated growth) and 220 µM (which repressed growth) for further investigation. Histological analysis of the bones treated with 40 µM dynasore revealed typical chondrocyte phenotype, with rounded cells in the RZ, columnar, flattened chondrocytes in the PZ, and enlarged cells in the HZ (Fig. 3A). By contrast, a high dose of dynasore (220 µM) led to empty lacunae in the RZ and PZ (Fig. 3A), suggesting potential cytotoxicity, which aligns with prior studies indicating that excessive disruption of dynamin activity can lead to cellular stress and apoptosis [16].

In order to assess changes in chondrocyte behavior that could explain the increase in bone growth caused by the 40 µM dynasore, we conducted histomorphometry. Firstly, we verified that bone growth had increased due to the effects of dynasore on chondrocytes, since the length of the epiphyseal cartilage was significantly increased in the presence of 40 µM dynasore in comparison with vehicle (Fig. 3A). Interestingly, greater differences were observed in the RZ and PZ (Fig. 3B) than the HZ height or in the height of the terminal hypertrophic cells (Fig. 3C). In contrast, bafilomycin stimulated elongation throughout the epiphyseal cartilage (Fig. 3B), whereas the reduced bone growth observed with the higher dose of dynasore (220 µM) was reflected in the reduced sizes of all parameters. To monitor changes in ECM turnover that could be mediated by dynamin-mediated endocytosis, we quantified the relative amount of ECM in the epiphysis. Interestingly, the all epiphyseal cartilage zones contained relatively more ECM in bones treated with dynasore at 40 µM than vehicle- or bafilomycin-treated bones (Fig. 3D-E).

### Low dose of dynasore decreased chondrocyte proliferation while maintaining collagen type X synthesis

**Fig. 4:**
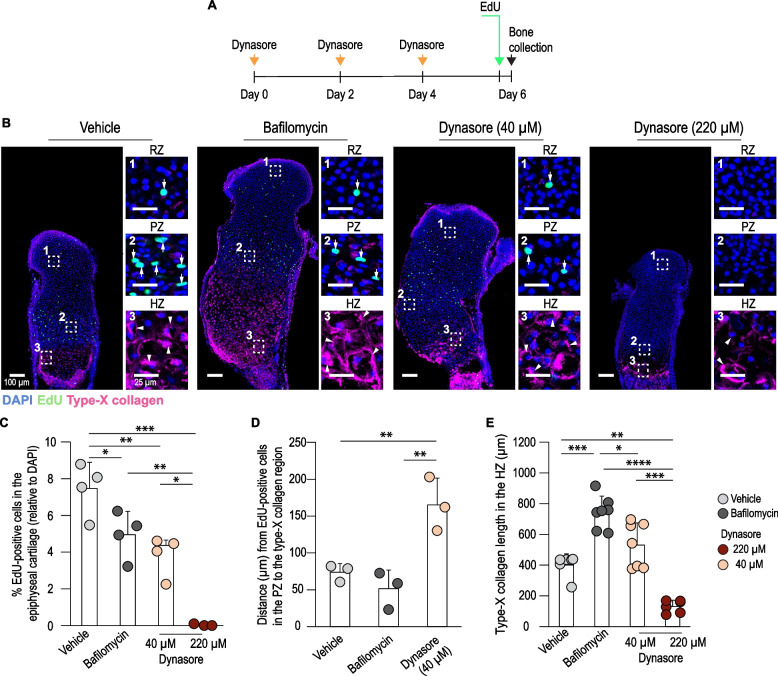
A low concentration of dynasore (40 µM) decreases chondrocyte proliferation while preserving collagen type X production. **A** Schematic representation of the bone cultures at day 6, as per Fig. 1C. EdU was added four hours before collecting the metatarsals at day 6. **B** EdU incorporation (white arrows in insets, visualized by Click assay) and collagen type X production (white arrowheads in insets, visualized by immunofluorescence) in chondrocytes. **C** Quantification of EdU-positive chondrocytes and **D** and distance from EdU-positive cells in the proliferative zone to the collagen type X-positive region. **E** Length of the collagen type X-positive region. RZ: resting zone; PZ: proliferative zone; HZ: hypertrophic zone. *N* = 4 bones obtained from one litter for C and *n* = 7 bones obtained from two litters for D, data is presented as mean ± SD. *p < 0.05, **p < 0.01, ***p < 0.001, and ****p < 0.0001. Please, note that the fluorescent images were obtained using maximum intensity projection

To better understand the cellular changes induced by dynasore, we labelled cells dividing in the last 4 h of culture, using the thymidine analogue, 5-ethynyl-2′-deoxyuridine (EdU) (Fig. 4A). Whereas vehicle-treated chondrocytes contained a relatively large proportion of dividing cells, all other treatments were associated with reduced cell division; this was most notable at the highest concentration (220 µM) of dynasore, which completely abolished chondrocyte proliferation as indicated by a total absence of EdU-positive cells (Fig. 4B-C). Even though the RZ and PZ were longer in dynasore 40 µM treatment, there was a reduced cell division rate in comparison to vehicle-treated bones. This was similar to bafilomycin treatment, in which a lower proportion of cells were proliferating despite an increased bone length (Fig. 4B-C) [15]. To further characterize the spatial relationship between proliferating and differentiating chondrocytes, we quantified the distance between EdU-positive cells and the region positive for HZ marker collagen type X (Fig. 4D), to measure the transition from the PZ to the HZ. Despite the marked reduction in EdU incorporation following dynasore treatment (40 µM), this distance was significantly increased compared to vehicle-treated controls. Together with our prior data indicating that dynasore (40 µM) caused ECM accumulation (Fig. 3D-E), these data demonstrate that the overall length of the cartilage, from the proliferating chondrocytes to the hypertrophic region was expanded by dynasore (40 µM).

To monitor the hypertrophic differentiation under the various culture conditions, the collagen type X immunostaining was quantified (Fig. 4B, E). Collagen type X-stained ECM likely represents a combination of remaining cartilage contained in the metatarsals at the point of tissue dissection as well as changes occurring during the culture period. Whilst bafilomycin stimulated the anticipated increase in chondrocyte hypertrophy in comparison to the vehicle-treatment [15], no significant changes were induced by dynasore at 40 µM. In contrast, exposure to 220 µM dynasore resulted in a marked reduction in collagen type X-stained region.

### Dynasore, at low concentrations, blocks autophagy and activates mTORC1 pathway in growing metatarsal bones

**Fig. 5:**
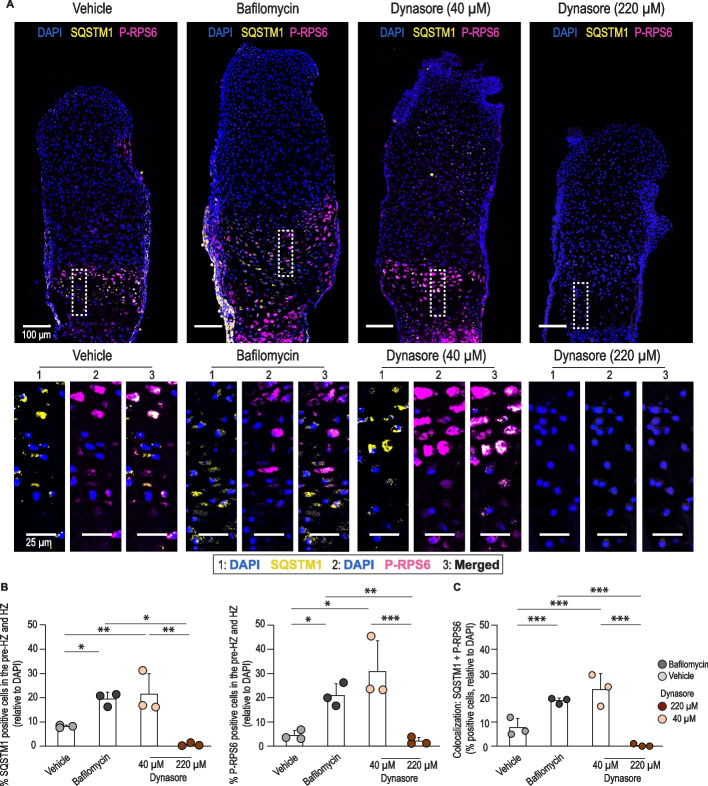
A low concentration of dynasore (40 µM) induces phosphorylation of RPS6 and accumulation of SQSTM1 in growing metatarsal bones. **A** Bones treated with 40 µM dynasore had accumulations of SQSTM1 and increased levels of phosphorylated-RPS6 in the epiphyseal cartilage, as visualized by immunofluorescence at day 6 of culture, as per Fig. 1C. **B**, **C** Quantifications of SQSTM1 and phosphorylated-RPS6 in the epiphysial cartilage. Each data point was obtained from an independent experiment containing 3 bones per group; data is presented as mean ± SD. *p < 0.05, **p < 0.01, ***p < 0.001. Fluorescent images were obtained using maximum intensity projection

One reported dynamin-independent target of dynasore is vacuolar-adenosine triphosphatase (v-ATPase), the specific target of bafilomycin. As there were similarities in metatarsal growth triggered by bafilomycin and dynasore (40 µM), we tested whether v-ATPase was inhibited in metatarsals following dynasore treatment. Since autophagy relies on lysosomal acidification by functional v-ATPase, the accumulation of the autophagy receptor, sequestosome 1 (SQSTM1), can be used as a readout of impaired v-ATPase function [15]. Interestingly, we found that a similar percentage of chondrocytes were positive for SQSTM1 in the presence of 40 µM dynasore as with bafilomycin, which were both significantly elevated in comparison with the vehicle (Fig. 5A-B). This was different from the perichondrium, in which there was more SQSTM1 accumulation in the bafilomycin group compared to the other groups (Additional file 1: Fig. S3A-B).

Bafilomycin treatment also triggers activation of ribosomal protein S6 (RPS6), a key readout of mTORC1 activity, in epiphyseal cartilage (Fig. 5A-C) [15]. We observed an increased phosphorylation of RPS6 in the pre-HZ and HZ at a low dose of dynasore (40 µM) and bafilomycin (Fig. 5A-C). This increase was also observed in the perichondrium of bafilomycin treatment (Additional file 1: Fig. S3). By contrast, both SQSTM1 and phosphorylated RPS6 were drastically decreased when higher doses of dynasore (220 µM) were used (Fig. 5A-C). These results indicate that 40 µM dynasore could block autophagy and activate mTORC1 signaling pathway in chondrocytes.

### Dynamin inhibition contributes to bone elongation

**Fig. 6:**
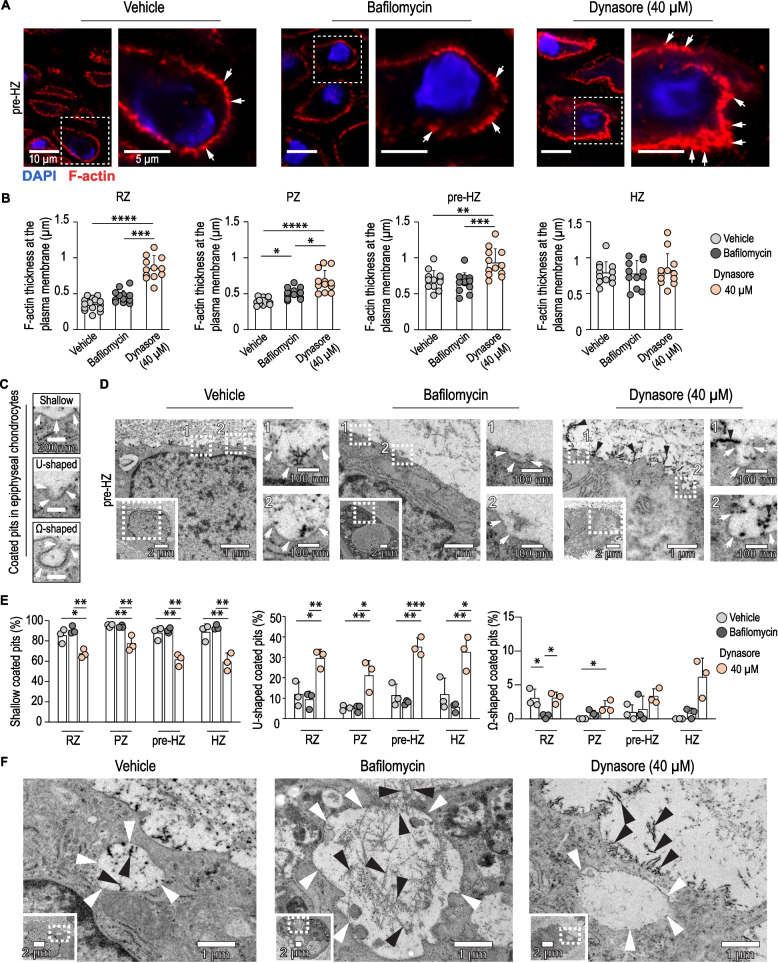
Endocytosis of cartilage matrix is impaired by dynasore. **A—B** Super-resolution microscopy images showing F-actin staining at the plasma membrane of pre-hypertrophic chondrocytes (white arrows) and quantification of the F-actin thickness in all zones. **C** Transmission electron microscopy (TEM) images of examples of shallow, U-shaped and Ω-shaped endocytic pits (white arrows) observed in the metatarsals. **D** Coated pits in pre-hypertrophic chondrocytes. Please, note the abundance of extracellular matrix (ECM) and fiber-like structures (black arrow heads) in the dynasore-treated group. **E** Quantification of shallow, U-shaped and Ω-shaped pits in the epiphysial cartilage. **F** TEM images reveal cartilage endocytosis in hypertrophic chondrocytes. Chondrocytes uptake collagen fibril-like electron-dense extracellular structures (black arrowheads) in endocytic-like pits/vesicles (white arrowheads). By contrast, dynasore-treated bones contained chondrocytes with over-accumulation of ECM in the extracellular space. RZ: resting zone; PZ: proliferative zone; HZ: hypertrophic zone. Each data point in B represent one cell whereas in E each data point is the average of three cells. All experiments are derived from 3 bones obtained from three different litters; data is presented as mean ± SD. *p < 0.05, **p < 0.01, ***p < 0.001 and ****p < 0.0001. Metatarsals were culture for 6 days, as per Fig. 1C

While our results indicated that dynasore and bafilomycin both stimulated longitudinal growth, only some of the underlying mechanisms were similar (namely, decreased chondrocyte proliferation; phosphorylated RPS6 and SQSTM1 levels) whereas others were different: bafilomycin stimulated hypertrophy (based on histomorphometry and collagen type X immunostaining), whereas 40 µM dynasore led to no change in cell size or hypertrophic differentiation but an increased distance from the proliferating chondrocytes to the HZ and the relative amount of ECM. To test whether alterations in endocytosis of material from the ECM could explain these differences, we first assessed changes in filamentous actin (F-actin) distribution, which is typically enriched at the plasma membrane in chondrocytes [17], and included pre-HZ to assess the process of hypertrophy. Interestingly, we noted a wider layer of F-actin at the cell perimeter of dynasore-treated bones; this pattern was present in RZ (Additional file 1: Fig. S4), PZ (Additional file 1: Fig. S4) and pre-HZ chondrocytes (Fig. 6A), while no changes were observed in cells that were already hypertrophic (Additional file 1: Fig. S4; Fig. 6B).

To verify if this disrupted F-actin distribution may reflect altered membrane trafficking, we visualized the ultrastructure of cell membranes using transmission electron microscopy (TEM). While endocytic pits (Fig. 6C) were evident on chondrocytes in the RZ (Additional file 1: Fig. S5A-B), PZ (Additional file 1: Fig. S5A-B), pre-HZ (Fig. 6DA-B) and HZ (Additional file 1: Fig. S5A-B), in all conditions, dynasore altered pit morphology: whereas shallower pits were predominantly found in vehicle and, particularly, bafilomycin-treated groups, dynasore caused pits to become deeper (Fig. 6E; Additional file 1: Fig. S5C). This observation is consistent with the role of dynamin in vesicle scission, and disruption of this process by dynasore preventing internalization of the material [6]. Furthermore, we also observed that dynasore-treatment caused endocytic pits to be coated with a more electron dense material (Fig. 6D). We used the TEM images to screen endosomal compartments for evidence of internalized ECM; structures resembling collagen fibril-like electron-dense extracellular structures were present (Fig. 6F).

Together, these data suggest that while bafilomycin promotes bone elongation by promoting chondrocyte hypertrophy (via blocking lysosomal v-ATPase, which activates mTORC1 signaling and the endosomal accumulation of endocytosed material due to impaired autophagy), dynasore stimulates longitudinal growth without promoting hypertrophy, which, despite also blocking lysosomal v-ATPase, appears to be caused by an accumulation of ECM in the extracellular space due to impaired cartilage remodeling.

To test if these differences could be directly attributed to the actions of dynasore on dynamin, we used an alternative dynamin inhibitor, dynole, that is structurally unrelated to dynasore [18]. Interestingly, metatarsal growth profiles resembled those with 40 µM dynasore: while a low dose (0.5 µM) dynole was able to stimulate growth, higher doses (> 10 µM) led to growth impairment (Additional file 1: Fig. S6A-C) (Additional file 2: Table S1 and Table S2). These data support the notion that the effects of dynasore on bone growth occur, at least in part, by dynamin-dependent mechanisms.

To understand if the effects of dynasore and bafilomycin were mutually exclusive, we assessed the effect of co-treatment on metatarsal growth (Additional file 1: Fig. S7). However, the simultaneous administration of dynasore and bafilomycin did not produce an additive or synergistic increase in bone length (Additional file 1: Fig. S7B-D). When used individually, higher concentrations of dynasore (Fig. 2B) and bafilomycin [24] both impair growth. We suspect that combining these doses produced a similar effect.

In summary, our findings indicate that at relatively low doses, prolonged use of dynasore can stimulate chondrocytes, causing tissue-wide effects that facilitate bone elongation, whereas at higher concentrations dynasore has deleterious effects, typical of hormesis.

## Discussion

As a small molecule, dynasore was identified as a potent inhibitor of the GTPase activity of dynamin-1 [6, 14] and subsequently was shown to potently inhibit dynamin function. However, further investigations revealed that it elicits effects in cells lacking dynamin genes [16] indicating dynamin-independent activities including altered cell membrane remodeling, destabilization of F-actin and v-ATPase inhibition [19]. Altogether, the use of dynasore to block dynamin becomes complicated by these multiple potential routes of action, which can be simultaneously affected and vary by cell type/state.

Using bafilomycin as the positive control for bone growth led us to directly compare the growth mechanisms with those of dynasore, which we thought could be similar as both are able to inhibit the v-ATPase. While we found some similarities with metatarsal growth induced by bafilomycin [15], specifically an increase in size of RZ + PZ length, reduced proliferation and elevated phosphorylated RPS6 and SQSTM1 accumulation, the effects were not identical since there was no marked increase in HZ parameters measured either by histology or collagen type X staining in the presence of dynasore. It is important to note that mTORC1 functions as a signaling hub integrating cues from multiple pathways to direct protein synthesis, and the effects of dynasore on the pathway reported above could be mediated through dynamin; this is plausible because growth factor receptor signaling is tightly coupled to endosomal trafficking, a process in which dynamin plays a critical role by regulating receptor presentation, signal attenuation, and degradation, for example [11, 20–24]. To determine whether the differential effects of dynasore on metatarsal growth were specifically linked to its inhibition of dynamin, we used dynole, a structurally distinct dynamin inhibitor. This parallel dose-dependent experimentation suggests that at least part of dynasore’s impact on bone growth is mediated through dynamin-dependent mechanisms, despite its known capacity to act on additional cellular targets. Future studies, to determine if changes in mTORC1 activity are due to alterations in receptor trafficking (i.e. may be dynamin-dependent) or v-ATPase inhibition (dynamin-independent) and further analysis of additional endosomal (e.g., early endosome antigen 1: EEA1) and lysosomal (e.g., lysosomal-associated membrane protein 1: LAMP1) markers, could provide complementary support to the present mechanistic interpretation.

In the presence of 40 µM dynasore, bone growth increased despite reduced cell proliferation and without a detectable change in hypertrophy. The relative amount of cartilage matrix as well as the distance between proliferating chondrocytes and collagen type X-stained cartilage both increased in the 40 µM dynasore group, indicated that metatarsal length was associated with extracellular matrix accumulation. By exploring cellular ultrastructures, we were able to identify collagen fibril-like electron-dense extracellular structures within endosomal compartments in the metatarsals. Since collagen fibril-like electron-dense extracellular structures are assembled in the extracellular space [25], it is highly likely that this material was being endocytosed rather than being retained in the cell via, for example, impaired exocytosis. This endocytosed material may contribute to the dry mass accumulation during hypertrophy of the chondrocytes [26], suggesting that dynamin-mediated endocytosis is needed for ECM remodeling and chondrocyte hypertrophy. Future studies using complementary methods, such as immunogold labeling or correlative light-electron microscopy, to confirm the identity of collagen fibers, as well as monitoring of protein synthesis rates, may be warranted to verify this possibility. Additionally, although dynasore inhibition resulted in increased growth throughout the six-day metatarsal culture period, this increase in length is likely transient, as a sustained reduction in proliferation rate and ECM accumulation would be unlikely to support continued bone elongation over longer timeframes.

Although we focused on the growth-promoting effects of dynasore at a dose of 40 µM, our results showed a hormetic effect: whereas low doses had a growth promoting effect, higher doses inhibited growth [8]. Hormetic responses, in which opposing effects occur at high and low doses of the same stimuli, are well described in the literature [8]. Given that dynasore has been shown to induce cell death [27], and nuclear abnormalities and empty lacunae were visible following six days with 220 µM dynasore treatment, dosing must be carefully considered. It was interesting to note that at doses safe to use in other tissues [9], 80 µM, for example, bone growth rates were very low after an initial stimulation of growth by day 2 of treatment.

Interestingly, dynasore may be safe to use to target chondrocytes in vivo. Indeed, dynasore administration to mice by intra-articular injection during osteoarthritis development, aiming to target articular chondrocytes, preserved cartilage integrity: fortnightly dynasore injections could reduce cartilage destruction (based on Osteoarthritis Research Society International “OARSI” score) caused by destabilization of the medial meniscus (DMM) surgery in both wild-type mice, and those with inducible chondrocyte-specific myosin light chain 3 mutations. The dynasore-induced amelioration was associated with a reduction in senescent markers p16^INK4A^ and γH2AX brought about by osteoarthritis (OA) development, which was attributed to reduced cartilage degradation via clathrin-mediated endocytosis [11]. The clear positive effects of treatment are interesting given the intermittent application of dynasore in this experiment, consistent with its application in other studies [10]. In the case that dynasore may be applied in vivo to test its ability to stimulate bone growth, establishing the optimal dose will be important to avoid the negative effects of over-exposure. Furthermore, long-term exposure must be carefully monitored since inhibiting cartilage endocytosis and turnover would inevitably impair continued growth. Hence, while dynasore is a valuable tool for studying dynamin-mediated processes, further research on its mechanisms of action, optimal concentration range, and potential off-target effects is needed to fully understand its hormetic influence on bone growth and to delineate the precise roles that dynamin plays during endochondral bone growth.

## Conclusions

In summary, our findings suggest that the dynamin inhibitor, dynasore, promotes longitudinal bone growth at low concentrations, whilst at higher concentrations it exerts deleterious effects.

## Methods

### Ex vivo culture of murine metatarsals

This system allows the study of chondrocytes at broad differentiation statuses within their intact three-dimensional tissue architecture, providing a more physiologically relevant model compared to monolayer or primary chondrocyte cultures [13, 15, 28]. After euthanasia, the hind paws of three-day-old C57BL/6 mice were collected, placed in 1.5 ml tubes (Sarstedt, catalog number: 72.690.001), and transported on ice to the animal cell laboratory. Then, under a stereo-microscope (Nikon SMZ645) and aseptically, the skin and soft tissues were gently removed followed by microdissection of the three central metatarsals from both hind paws using fine forceps (VWR, catalog number: OUTI5-DURAX). Microdissection was performed in pre-cooled (4 °C) DMEM/F-12 (Dulbecco's Modified Eagle Medium/Nutrient Mixture F-12) with GlutaMAX™ (Gibco™, catalog number: 31331093) and supplemented with 50 mg/ml gentamycin (Gibco™, catalog number: 15750045). Metatarsals were cultured for up to six days in DMEM/F-12 medium supplemented with 1 mM β-glycerophosphate (BGP) (Sigma-Aldrich, catalog number: G9422), 50 µg/ml L-ascorbate-2-phosphate (ascorbic acid) (Sigma-Aldrich, catalog number: G5960), 0.2% bovine serum albumin (Sigma-Aldrich, catalog number: 05470) and 20 mg/ml gentamycin, as previously reported [15]. Bone cultures were maintained in an incubator (37 °C, 5% CO_2_) and the medium was changed every two days. Three central metatarsals micro-dissected from one paw were used as controls (vehicle-treated) whereas the three central metatarsals from the contralateral paw were used as the experimental group (treatment). Therefore, each observation is the average of all three bones from one paw (i.e. one observation = one animal), unless otherwise stated in the figure legend. Please note that the distal end of the metatarsal was used for the histological analyses presented. To evaluate potential sex differences in response to dynasore, murine sex was determined by assessing anogenital pigmentation, as recently described [29]. This was verified by dissecting and visualizing the gonads using stereoscopic microscopy. These data (Additional file 1: Fig. S2) were then plotted separately from other experiments describe below.

### Metatarsal treatment with dynasore, dynole and EdU

Under sterile conditions, dynasore (Sigma-Aldrich, catalog number: 324410) was prepared as a 20 mM stock solution in DMSO (Sigma-Aldrich, catalog number: D8418), aliquoted into 20 µl portions, and stored at −20 °C in the dark until further use. Ex vivo metatarsal cultures, as described in Ex vivo* culture of murine metatarsals*, were treated with dynasore (at concentrations of 40 µM, 80 µM, 160 µM, and 220 µM) on days 0, 2, and 4. Bafilomycin (8 nM; Thomas Scientific, catalog number: C974N94), a well-established compound known to induce overgrowth in tubular bones [15], was used as a positive control, and also used in combination with 40 µM dynasore. EdU (ThermoFisher, catalog number: A20012) was prepared in distilled water (Invitrogen, catalog number: 10977–035) as a 5 mM stock solution and kept at −20 °C. Subsequently, EdU at 5 µM was added to the metatarsal cultures 4 h before collecting the bones at day 6. To evaluate the effect of structurally distinct dynamin inhibitors, we use dynole in a similar dose response as previously described: 0.2 µM, 0.5 µM, 1 µM, 10 µM, 20 µM, and 40 µM [30]. Please, note that dynole was prepared following the manufacturer’s instructions. Briefly, dynole was diluted in DMSO as a 100 mM stock solution and stored at −20 °C in the dark until further use.

Longitudinal bone growth was documented every two days (days 0, 2, 4, and 6) using an Infinity 1 camera integrated with a Nikon SMZ-U stereo-microscope. Bone length measurements were subsequently performed using ImageJ analysis software (National Institutes of Health) from the tip of the proximal epiphysial cartilage to the tip of the distal one, with curvature taken into consideration as described [13].

### Safranin O/Fast Green staining of metatarsals

Metatarsals were fixed in 4% paraformaldehyde (PFA, prepared by diluting formaldehyde [Sigma-Aldrich, catalog number: 8.18708.1000] in 1X PBS) for 48 h, then transferred to 10% ethylenediaminetetraacetic acid (EDTA, pH 8.05; Scharlau, catalog number: AC09401000) for 48 h followed by 70% ethanol (EtOH). These incubations were carried out under gentle shaking on a rotating platform (IKA, KS-130 basic) at 4 °C. Next, bones underwent dehydration overnight in a LOGOS Microwave Hybrid Tissue Processor (Milestone). The dehydrated samples were embedded in paraffin and sectioned at a thickness of 5 µm using a microtome (Marshall Scientific, Microm HM 360). The 5 µm sections were floated on heated autoclaved water (60 °C) to remove wrinkles, and then mounted on Epredia™ SuperFrost Plus™ Gold slides (ThermoFisher, catalog number: 11976299). Thereafter, sections were incubated at 60 °C for 1 h in a Hybaid oven to remove paraffin, followed by sequential rehydration through xylene (twice), 99% EtOH, 95% EtOH, and 70% EtOH, with each step lasting 5 min. The samples were then stained with Weigert's hematoxylin solution, prepared with hematoxylin (Sigma-Aldrich, catalog number: H3136) and ferric chloride (Sigma-Aldrich, catalog number: 157740), for 5 min, rinsed in distilled water for 3 min, briefly dipped (2 s) in acid alcohol, and washed again with distilled water. Next, the slides were stained with 0.02% Fast Green (Sigma-Aldrich, catalog number: F7258) for 3 min, treated with 1% acetic acid for 3 min, and, without rinsing, stained with Safranin O solution (Sigma-Aldrich, catalog number: S8884) for 30 min. Following staining, the samples were rinsed in 95% EtOH and dehydrated through sequential steps of 95% EtOH, 99% EtOH (twice), and xylene (twice), each step lasting 5 min. Finally, the sections were mounted using Pertex mounting medium (Histolab, catalog number: 00811) and imaged using a Zeiss AX10 microscope equipped with an Axiocam MRm camera.

Please note that, for histomorphometry analyses, we have defined the epiphyseal cartilage zones based on our prior reports [1]: RZ: defined as the region containing round-like, scattered chondrocytes with no evident of columnar organization. PZ: identified by flattened chondrocytes arranged in longitudinal columns parallel to the axis of growth, consistent with actively dividing transit-amplifying cells. HZ: defined as the region containing enlarged (hypertrophic), rounded chondrocytes with increased cell volume and lacunar size. Boundary between PZ and HZ (also known as pre-hypertrophic zone; pre-HZ): operationally defined as the point at which chondrocytes transition from being twice as wide as they were high towards enlarged, rounded cells.

Histomorphometric analyses of the epiphyseal cartilage zones, as well as hypertrophic cell height were then performed using ImageJ. Please note that for the histomorphometry analysis we merged the RZ and PZ. To determine the length of each zone, ten straight lines were drawn across the region of interest parallel to the axis of growth, and averaged to obtain a single value per bone. The relative amount of matrix was also quantified using ImageJ software. Briefly, images were converted to 8-bit format, the epiphyseal cartilage boundary (including either “RZ + PZ” or “HZ”) was delineated, and a Gaussian blur filter (radius = 2) was applied before intensity thresholding. The matrix content was then calculated as the percentage of the total area. Furthermore, using these settings and applying intensity thresholding, we generated images that reveal the extent of the ECM surrounding the lacunae in which chondrocytes are embedded in the epiphyseal cartilage.

### Multiplex immunofluorescence for phospho-RPS6 and SQSTM1

Bone sections were prepared on glass slides, incubated at 60 °C to remove paraffin and then rehydrated, following the protocol outlined in *Safranin O/Fast Green staining of metatarsals*. Next, the specimens were placed in DAKO target retrieval buffer (Agilent technologies, catalog number: S1699) and heated in a sealed pressure cooker (CertoClav A-4050) at 0.2 bar. After cooling the pressure cooker for 30 min, the samples were removed and rinsed three times with distilled water and washed once with phosphate buffered saline (PBS, 1X) containing 0.05% Tween 20 (PBST; Sigma-Aldrich, catalog number: p9416), hereafter known as PBST. Please note that the samples were outlined with an ImmEdge™ pen (Vector Laboratories, catalog number: H-4000) prior to incubation with PBST, thus ensuring that the samples were fully immersed in every solution during the immunostaining procedure. Blocking was performed using 3% normal horse serum (Jackson ImmunoResearch, catalog number: 8000121) diluted in PBST with 0.05% Triton 100X (Sigma-Aldrich, catalog number: T8787) for 1 h at room temperature. Primary antibodies targeting phospho-RPS6 (1:100; Cell Signaling, catalog number: 4858) and SQSTM1 (1:500; Progen, catalog number: GP62-C) were diluted in the blocking buffer and incubated with the samples overnight at 4 °C. The following day, the slides were equilibrated to room temperature for 15 min and washed three times with PBST, each wash lasting 5 min. Secondary antibodies (1:400) were prepared in blocking buffer and applied to the samples for 1 h at room temperature. For phospho-RPS6, the secondary antibody was purchased from Invitrogen (catalog number: A31573), and for SQSTM1, it was acquired from Jackson ImmunoResearch Laboratories (catalog number: 706–545-148). After secondary antibody incubation, slides were rinsed once with PBST for 5 min. Subsequently, the sections were stained with DAPI (1.5 µg/ml; Sigma-Aldrich, catalog number: D9542) diluted in PBS (1X) for 15 min, followed by three washes with PBS (1X). The samples were mounted using Fluoroshield mounting medium (Sigma-Aldrich, catalog number: F6182) and imaged using a Leica Stellaris 5 X advanced line scanning confocal microscope. To enhance accessibility, all fluorescence images were generated using a color-blind–friendly palette. Briefly, samples were imaged using a 20 × objective with a pixel size of 581.25 µm × 581.25 µm and a format of 1024 × 1024. Potential under- or overexposure of the samples was checked and adjusted prior to image acquisition to enhance image quality. Subsequently, imaging was performed at a speed of 400 with a line average of 1, and Z-stacks were acquired at 2.05 µm intervals. Laser power was set to 10% for DAPI, 20% for Alexa-Fluor 488 (SQSTM1), and 30% for Alexa-Fluor 647 (phospho-RPS6). Z-stack images were processed as maximum intensity projections using LAS X software (Leica Mycrosystems, version 1.4.6.28433). Quantification of positive cells for phospho-RPS6 and SQSTM1, normalized to DAPI positive cells, were performed using QuPath software (version 0.5.1) [31]. For this, images were uploaded on the QuPath software opened as fluorescence type, and the pixel size was adjusted to 0.3 µm, thus ensuring compatibility with the cell detection settings. Further, the full epiphyseal cartilage or the perichondrium were separately annotated followed by cell quantification for DAPI, green (SQSTM1) or red (phospho-RPS6) channels. Please note that all quantitative analyses were performed using single-channel measurements, with the exception of the co-localization assessments, for which signal overlap between the red and green channels was evaluated at the single-cell level using QuPath’s measurement functions to determine the proportion of cells exhibiting simultaneous phospho-RPS6 and SQSTM1 fluorescence.

### Immunostaining for collagen type X multiplexed with EdU staining

Bones were fixed in 4% PFA for six hours, followed by overnight incubation in 30% sucrose solution (diluted in 1X PBS) (AG Scientific, catalog number: S-2885) on a rotating platform at 4 °C. The following day, bones were embedded in optimal cutting temperature (OCT) medium (Sakura Finetek, catalog number: 4583) and frozen on dry ice. Cryosectioning was performed at a thickness of 20 µm using an NX70 Cryostat (Epredia) and sections were placed on Epredia™ SuperFrost Plus™ Gold slides. The samples were air-dried and stored at −20 °C until further use. Prior to staining, the sections were thawed at room temperature and air-dried. The samples were then incubated in PBS (1X) for 15 min in a humidified chamber. Subsequently, blocking was performed using 3% normal horse serum diluted in PBST containing 0.25% Triton X-100. Collagen type X primary antibody (1:750; Abcam, catalog number: 58632) was diluted in the blocking buffer and incubated with the samples overnight at 4 °C. Subsequently, the slides were washed three times with PBST for five minutes each, incubated with a secondary antibody (1:600; Invitrogen, catalog number: A31572) at room temperature and washed three times with PBST, each wash lasting 5 min. Simultaneously, the EdU reaction mix was prepared in a chemical hood. This mix contained 1 M TRIS (pH 7.5), 100 mM CuSO_4_, Alexa-Azide 647 (diluted 1:10) (Thermo Fisher Scientific, catalog number: A10277), distilled water, and 0.5 M ascorbic acid (diluted in distilled water). Samples were incubated with the reaction mix for 30 min, followed by a five-minute rinse with PBST. Nuclei were counterstained with DAPI for 15 min, and the samples were mounted using Fluoroshield, as described in *Multiplex immunofluorescence for phospho-RPS6 and SQSTM1*. Imaging was performed using a Leica Stellaris 5 X confocal microscope and quantifications of EdU-positive cells (relative to DAPI) and length of collagen type X were processed with QuPath software, as described in *Multiplex immunofluorescence for phospho-RPS6 and SQSTM1*. Please, note that for EdU quantification, we included the full epiphyseal cartilage in the analysis, as we previously reported [15], thus showing the overall proliferation rate in this tissue. Additionally, we measured the distance from every EdU-positive cell in the proliferating zone to the nearest part of type-X collagen positive in the hypertrophic zone using ImageJ. Epiphyseal growth plate zones were define following the criteria in *Safranin O/Fast Green staining of metatarsals*.

### F-actin staining and super-resolution microscopy

Metatarsals were cultured under vehicle, bafilomycin or dynasore (40 µM) and processed for fixation, OCT embedding and cryo-sectioning as in *Safranin O/Fast Green staining of metatarsals* and *Immunostaining for collagen type X multiplexed with EdU staining* respectively. Then, tissue sections were incubated with PBS (1X) for 15 min in a humidified chamber. Subsequently, bones were incubated simultaneously with Alexa Fluor 647-conjugated phalloidin (Thermo Fisher Scientific, Cat. No. A22287) to stain filamentous actin (F-actin), and DAPI to label nuclei, for 45 min at room temperature. After staining, samples were rinsed three times with 1X PBS and mounted using Fluoroshield. Fluorescence images were acquired using a Zeiss LSM 980 confocal microscope equipped with an Airyscan 2 detector. Sequential imaging was performed using appropriate laser lines and filter settings for Alexa Fluor 647 and DAPI. Airyscan super-resolution mode was used where indicated. Briefly, samples were imaged using a 63 × immersion objective with oil (Immersol 518. ISO 8036–1/2, ne = 1.518, 23 °C, halogen-free. Carl Zeiss Microscopy, catalog number: 444964–0000-000) and a pixel size of 0.035 µm × 0.035 µm and 8-bit depth. Laser excitation was set to 0.5% for both 639 nm (F-actin) and 405 nm (DAPI). Images were acquired in bidirectional scan mode with a line step of 1, averaging of 8, detector gain of 850 V, and detector digital gain of 1. All images were captured using the snap mode, and no further processing, such as maximum intensity projection or deconvolution, was performed. Images were subsequently analyzed using ImageJ to measure the thickness of the F-actin staining around the inner leaflet of the plasma membrane, based on a 2D line [32]. Briefly, images were thresholded and converted to mask. Then a line was drawn using the longest edge containing F-actin. The width of F-actin staining was measured at each end of the line. Thus, two measurements were collected per cell, which were subsequently averaged. Epiphyseal growth plate zones were define following the criteria in *Safranin O/Fast Green staining of metatarsals*.

### Transmission electron microscopy (TEM)

Metatarsals were cultured under vehicle, bafilomycin or dynasore (40 µM) as in *Metatarsal treatment with dynasore, dynole and EdU*. Bone specimens were promptly fixed in a solution containing 2.5% glutaraldehyde and 1% PFA, prepared in 0.1 M Sorensen phosphate buffer (pH 7.4) at room temperature for 1 h followed by storage at 4 °C. The metatarsals were then rinsed in 0.1 M sodium phosphate buffer (pH 7.4) and post-fixed in 2% osmium tetroxide in the same buffer at 4 °C for 2 h. Dehydration was carried out through a graded ethanol and acetone series, after which the specimens were embedded in LX-112 epoxy resin (Ladd Research). Ultrathin sections (~ 80—100 nm) were prepared using a Leica EM UC7 ultramicrotome, transferred onto formvar slot grids stained sequentially with uranyl acetate and lead citrate, and imaged using a Hitachi HT7800 transmission electron microscope operating at 100 kV. Digital micrographs were captured with a 20MPx Xarosa CMOS camera (EMSIS GmbH).

### Endocytic coated pits analysis

For morphometric analysis, only cells with a fully visible perimeter in the electron micrographs were included as previously reported [33]. Briefly, endocytic coated pits were categorized based on their morphology observed in transmission electron micrographs, following well established ultrastructural criteria for clathrin-coated structures as reported by Nández and colleagues [33]. Using this prior work and calibrated TEM images in ImageJ, each coated profile along the plasma membrane was examined and manually classified into one of three categories: (1) shallow pits: slight invaginations with a visible clathrin coat but minimal curvature; (2) U-shaped pits: more pronounced invaginations; and 3) Ω-shaped pits still continuous with the plasma membrane. This allowed consistent distinction between early, intermediate, and late stages, respectively, of coated pit formation. Please note that our identification of endocytic pits was also supported by considering their spatial distribution, size, and frequent association with vesicular structures immediately beneath the membrane, as reported previously [33]. All these endocytic coated structures (shallow, U-shaped and Ω-shaped) were quantified in the RZ, PZ, pre-HZ, and HZ. Additionally, the pit width/depth ratio was also quantified using ImageJ. Briefly, lines were drawn across each pit using the ‘Straight Line’ tool in ImageJ. Pit width was measured as the horizontal distance between the pit edges, and pit depth as the vertical distance from this line to the pit’s lowest point. The width/depth ratio was then calculated, with higher values indicating shallower pits and lower values indicating deeper pits. Epiphyseal growth plate zones were define following the criteria in *Safranin O/Fast Green staining of metatarsals*.

### Statistics and reproducibility

To increase the robustness and reproducibility of the data, every observation within each treatment group derives from a mouse obtained from a different litter, unless otherwise stated in the figure legend. Bone length measurements (Fig. 2B-C; Additional file 1: Fig. S1B; Additional file 1: Fig. S2B-C), histomorphometry (Fig. 3B and E) and collagen type X length staining (Fig. 4B-C) and pit width/depth ratio quantifications (Additional file 1: Fig. S5C) were performed in a single-blinded manner.

When the assumption of normality was met (using the Shapiro–Wilk test), a statistical analysis was conducted using one-way ANOVA followed by Tukey’s multiple comparison test to evaluate differences between experimental groups, with significance levels denoted as *p < 0.05, **p < 0.01, ***p < 0.001, and ****p < 0.0001. When the assumption of normality was not met (Fig. 2C, day 6; Fig. 3B: hypertrophic cell height; Fig. 6B: RZ, PZ, and pre-HZ; Additional file 1: Fig. S5C: chondrocytes pits width/depth ratio; Additional file 1: Fig. S5B: number of shallow pits (RZ), number of U-shaped pits (RZ, PZ, pre-HZ and HZ), number of Ω-shaped pits (RZ, PZ, pre-HZ and HZ); Fig. S7A-B: day 2), the Kruskal–Wallis test was used instead, followed by Dunn’s test for multiple comparisons, with the same significance thresholds. For comparison between two groups, a Welch Two Sample t-test was applied (Additional file 1: Fig. S2B-C). Graphs present data as mean ± SD, with individual data points included where appropriate. Details of data normalization are provided in the figure legends. All statistical analyses were performed using RStudio software (version 4.1.2, 2021–11-01) and graphs produced in Excel (2019 MSO. 16.0.10416.20047). Schematics were made with Biorender.com (agreement numbers: BY285AJ4ZS and ZV285AIZB6) and Illustrator (version 29.4. Adobe Creative Cloud).

## Supplementary Information

Additional file 1: Fig. S1 – S7. Fig. S1. Longitudinal bone growth of metatarsal ex vivo cultures from day 0 to day 6, as per Fig. 1C. A Microphotographs of metatarsal bones were collected during culture. B Bone growth rate. Data show three independent experiments per group and is presented as mean ± SD. **p < 0.01. Fig. S2. *Ex vivo* assessment of sex-dependent bone responses to dynasore in mice. A Microphotographs of metatarsals at day 6 of *ex vivo* culture, as per Fig. 1C. Bone growth rate in males and females. Data show three independent experiments per group and is presented as mean ± SD. *p < 0.05. **p < 0.01. ***p < 0.001. Fig. S3. Quantifications of SQSTM1 and phosphorylated-RPS6 and colocalization in the perichondrium. Data represent three independent experiments per group and is presented as mean ± SD. *p < 0.05. **p < 0.01. Fig. S4. Super-resolution microscopy images showing F-actin staining at the plasma membrane of epiphyseal chondrocytes. N = 3 bones from three different litters. Bones were kept in culture for six days, as per Fig. 1C. Fig. S5. Analysis of endocytic pits. A, B Transmission electron microscopy images of epiphyseal chondrocytes and quantification of shallow pits, U-shaped pits and Ω-shaped pits, as described in Fig. 6C. C chondrocytes pit width/depth ratio. Please note that insets in A show the border of different forms of coated pits, where pit peripheries are indicated by black arrows. Each data point represents a cell in B. All experiments are derived from 3 bones obtained from a different litter each. Metatarsals were cultured for six days, as per Fig. 1C. Data is presented as mean ± SD. *p < 0.05. **p < 0.01. ***p < 0.001. ****p < 0.0001. Fig. S6. Dynole directs differential regulation of longitudinal bone growth. A, B Longitudinal bone growth was assessed every two days. n = 4 animals for vehicle and bafilomycin; n = 3 animals for 0.2 µM, 0.5 µM, 10 µM, 20 µM and 40 µM dynole; n = 6 for 1 µM. Asterisks in panel B, C indicate statistically significant differences assessed by one-way ANOVA. Data is presented as mean ± SD. *p < 0.05. **p < 0.01. ***p < 0.001. ****p < 0.0001. Please note that whereas Day 2 has all the p-values represented in C, the full list of p-values obtained for Day 4 and Day 6 are available in Table S1 and Table S2, respectively. Bones were cultured for 6 days, as per Fig. 1C. Fig. S7. Bafilomycin and dynasore co-administration does not additively or synergistically enhance bone growth. A Schematic representation of the experimental approach. B Representative images at day 6 of metatarsals ex vivo cultures, as per Fig. 1C. Bones were maintained in the presence of vehicle, bafilomycin, dynasore, and dynasore + bafilomycin. C, D Longitudinal bone growth was assessed every two days. n = 3 bones for vehicle, bafilomycin and dynasore, except for dynasore+ bafilomycin which has an n = 6 bones. Data is presented as mean ± SD. *p < 0.05. **p < 0.01. ***p < 0.001.

Additional file 2: Table S1. Full list of p-values generated in the statistical analysis of dynole at day 4 of culture presented in Fig. S6. * represents *p* < 0.05, **p < 0.01, ***p < 0.001, ****p < 0.0001, and “ns” = not significant. Table S2. Full list of p-values generated in the statistical analysis of dynole at day 6 of culture presented in Fig. S6.* represents *p* < 0.05, **p < 0.01, ***p < 0.001, ****p < 0.0001, and “ns” = not significant.

## Data Availability

The underlying data for this study are available upon request.

## Abbreviations

DMM: Medial meniscus
ECM: Extracellular matrix
EdU: 5-Ethynyl-2′-deoxyuridine
EEA1: Early endosome antigen 1
F-actin: Filamentous actin
FGF: Fibroblast growth factor
GTPase: Guanosine triphosphatase
GH: Growth hormone
HZ: Hypertrophic zone
IGF-1: Insulin-like growth factor 1
IHH: Indian hedgehog
LAMP1: Lysosomal-associated membrane protein 1
mTORC1: Mechanistic target of rapamycin complex 1
OARSI: Osteoarthritis Research Society International
OA: Osteoarthritis
pre-HZ: Pre-hypertrophic zone
POC: Primary ossification center
P-RPS6: Phosphorylated ribosomal protein S6
PTHrP: Parathyroid hormone-related peptide
PZ: Proliferative zone
RPS6: Ribosomal protein S6
RZ: Resting zone
SQSTM1: Sequestosome 1
TEM: Transmission electron microscopy
v-ATPase: Vacuolar-adenosine triphosphatase
