# Supplementary material for: Dynamin inhibitor dynasore modulates longitudinal bone growth in a hormetic manner
Source: BMC Biol. 2026 Jul 16;24:169. doi: 10.1186/s12915-026-02687-4 (PMC13374082; doi:10.1186/s12915-026-02687-4)
Supplement: Supplementary file 2 — Additional file 2: Table S1. Full list of p-values generated in the statistical analysis of dynole at day 4 of culture presented in Fig. S6. * represents p < 0.05, **p < 0.01, ***p < 0.001, ****p < 0.0001, and “ns” = not significant. Table S2. Full list of p-values generated in the statistical analysis of dynole at day 6 of culture presented in Fig. S6.* represents p < 0.05, **p < 0.01, ***p < 0.001, ****p < 0.0001, and “ns” = not significant. [file 12915_2026_2687_MOESM2_ESM.docx]

**Table S1.** Full list of p-values generated in the statistical analysis of dynole at day 4 of culture presented in Fig. S6. * represents p < 0.05, **p < 0.01, ***p < 0.001, ****p < 0.0001, and “ns” = not significant.

|  |  | Vehicle | Bafilomycin | Dynole concentration | | | | | |
| --- | --- | --- | --- | --- | --- | --- | --- | --- | --- |
|  |  |  | 0.2 µM | 0.5 µM | 1 µM | 10 µM | 20 µM | 40 µM |  |
| Vehicle | | / | **** | ns | * | ns | * | *** | **** |
| Bafilomycin | | **** | / | *** | * | *** | **** | *** | **** |
| Dynole concentration | 0.2 µM | ns | *** | / | ns | ns | * | * | *** |
|  | 0.5 µM | * | * | ns | / | ns | ns | *** | **** |
|  | 1 µM | ns | *** | ns | ns | / | ** | *** | **** |
|  | 10 µM | * | **** | * | ns | ** | / | ns | ns |
|  | 20 µM | *** | *** | * | *** | *** | ns | / | ns |
|  | 40 µM | **** | **** | *** | **** | **** | ns | Ns | / |

|  |  | Vehicle | Bafilomycin | Dynole concentration | | | | | |
| --- | --- | --- | --- | --- | --- | --- | --- | --- | --- |
|  |  |  |  | 0.2 µM | 0.5 µM | 1 µM | 10 µM | 20 µM | 40 µM |
| Vehicle | | / | *** | ns | ns | ns | ** | *** | **** |
| Bafilomycin | | *** | / | *** | * | *** | **** | **** | **** |
|  | 0.2 µM | ns | *** | / | ns | ns | ** | ** | *** |
|  | 0.5 µM | ns | * | ns | / | ns | ns | **** | **** |
| Dynole | 1 µM | ns | *** | ns | ns | / | *** | *** | *** |
| concentration | 10 µM | ** | **** | ** | ns | *** | / | ns | ns |
|  | 20 µM | *** | **** | ** | **** | *** | ns | / | ns |
|  | 40 µM | **** | **** | *** | **** | *** | ns | ns | / |

**Table S2.** Full list of p-values generated in the statistical analysis of dynole at day 6 of culture presented in Fig. S6. * represents p < 0.05, **p < 0.01, ***p < 0.001, ****p < 0.0001, and “ns” = not significant.
