# Supplementary material for: Dynamin inhibitor dynasore modulates longitudinal bone growth in a hormetic manner
Source: BMC Biol. 2026 Jul 16;24:169. doi: 10.1186/s12915-026-02687-4 (PMC13374082; doi:10.1186/s12915-026-02687-4)
Supplement: Supplementary file 1 — Additional file 1: Fig. S1 – S7. Fig. S1. Longitudinal bone growth of metatarsal ex vivo cultures from day 0 to day 6, as per Fig. 1C. A Microphotographs of metatarsal bones were collected during culture. B Bone growth rate. Data show three independent experiments per group and is presented as mean ± SD. **p < 0.01. Fig. S2. Ex vivo assessment of sex-dependent bone responses to dynasore in mice. A Microphotographs of metatarsals at day 6 of ex vivo culture, as per Fig. 1C. Bone growth rate in males and females. Data show three independent experiments per group and is presented as mean ± SD. *p < 0.05. **p < 0.01. ***p < 0.001. Fig. S3. Quantifications of SQSTM1 and phosphorylated-RPS6 and colocalization in the perichondrium. Data represent three independent experiments per group and is presented as mean ± SD. *p < 0.05. **p < 0.01. Fig. S4. Super-resolution microscopy images showing F-actin staining at the plasma membrane of epiphyseal chondrocytes. N = 3 bones from three different litters. Bones were kept in culture for six days, as per Fig. 1C. Fig. S5. Analysis of endocytic pits. A, B Transmission electron microscopy images of epiphyseal chondrocytes and quantification of shallow pits, U-shaped pits and Ω-shaped pits, as described in Fig. 6C. C chondrocytes pit width/depth ratio. Please note that insets in A show the border of different forms of coated pits, where pit peripheries are indicated by black arrows. Each data point represents a cell in B. All experiments are derived from 3 bones obtained from a different litter each. Metatarsals were cultured for six days, as per Fig. 1C. Data is presented as mean ± SD. *p < 0.05. **p < 0.01. ***p < 0.001. ****p < 0.0001. Fig. S6. Dynole directs differential regulation of longitudinal bone growth. A, B Longitudinal bone growth was assessed every two days. n = 4 animals for vehicle and bafilomycin; n = 3 animals for 0.2 µM, 0.5 µM, 10 µM, 20 µM and 40 µM dynole; n = 6 for 1 µM. Asterisks in panel B, C indicate statistic [file 12915_2026_2687_MOESM1_ESM.zip › Fig S3.pdf]

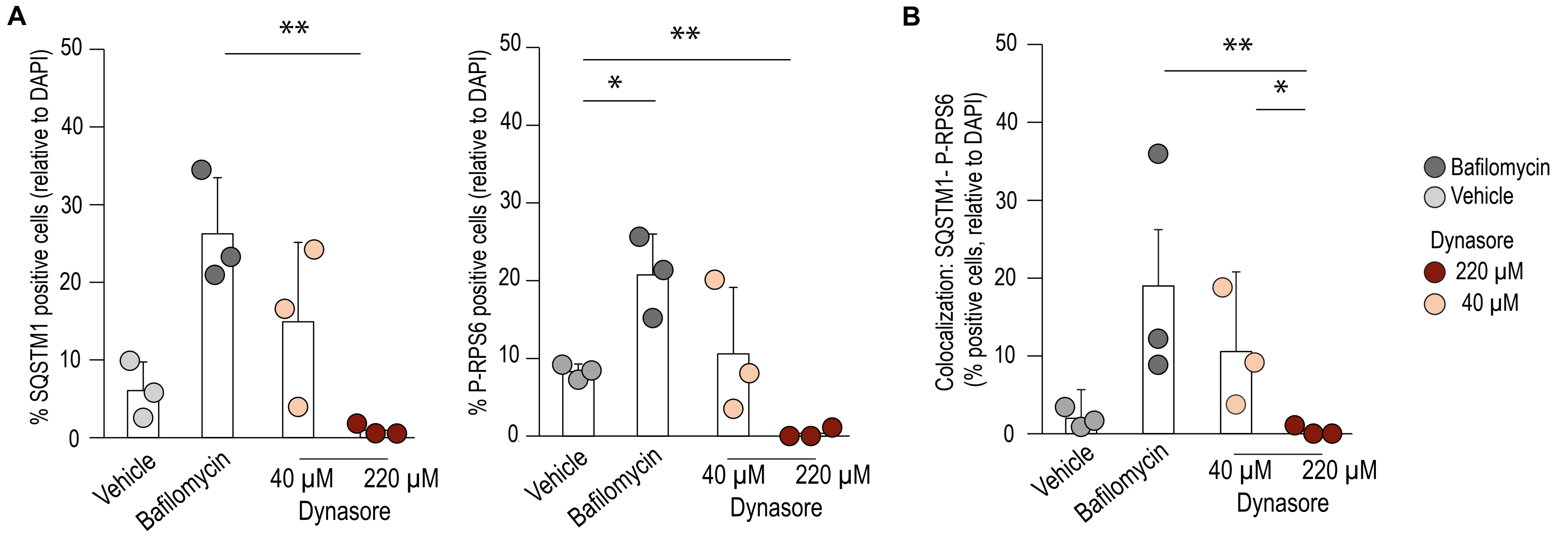

A
% SQSTM1 positive cells (relative to DAPI)
50
40
30
20
10
0
**
Vehicle
Bafilomycin
40 µM
220 µM
Dynasore
% P-RPS6 positive cells (relative to DAPI)
**
*
B
Colocalization: SQSTM1- P-RPS6
(% positive cells, relative to DAPI)
Bafilomycin
Vehicle
Dynasore
220 µM
40 µM
